# *Notes from the Field*: Occupational Lead Exposures at a Shipyard — Douglas County, Wisconsin, 2016

**DOI:** 10.15585/mmwr.mm6601a8

**Published:** 2017-01-13

**Authors:** Debora Weiss, Stephanie J. Yendell, Luke A. Baertlein, Krista Y. Christensen, Carrie D. Tomasallo, Paul D. Creswell, Jenny L. Camponeschi, Jon G. Meiman, Henry A. Anderson

**Affiliations:** ^1^Epidemic Intelligence Service, CDC; ^2^Wisconsin Department of Health Services, Division of Public Health, Bureau of Environmental and Occupational Health; ^3^Minnesota Department of Health, Health Risk Intervention Unit; ^4^Department of Population Health Sciences, University of Wisconsin-Madison.

On March 28, 2016, the Minnesota Poison Control System was consulted by an emergency department provider regarding clinical management of a shipyard worker with a blood lead level (BLL) >60 *μ*g/dL; the National Institute for Occupational Safety and Health defines elevated BLLs as ≥5 *μ*g/dL (*1*). The Minnesota Poison Control System notified the Minnesota Department of Health (MDH). Concurrently, the Wisconsin Department of Health Services (WDHS) received laboratory reports concerning two workers from the same shipyard with BLLs >40 *μ*g/dL. These three workers had been retrofitting the engine room of a 690-foot vessel since January 4, 2016.

Work was suspended during March 29–April 4 in the vessel’s engine room, the presumptive primary source of lead exposure. On March 29, the shipyard partnered with a local occupational health clinic to provide testing for workers. Employees and their household members were also tested by general practitioners and local laboratories. The shipyard hired sanitation crews for lead clean-up and abatement and provided personal protective equipment for its employees. On April 1, WDHS and MDH issued advisories to alert regional health care organizations, local public health agencies, and tribal health departments to the situation and launched a joint investigation on April 4. Subsequently, WDHS activated its Incident Command System and worked with MDH to compile a list of potentially exposed workers. By August 31, a total of 357 workers who might have been employed at the shipyard during December 2015–March 2016 had been identified.

During April–July 2016, WDHS and MDH attempted telephone interviews with workers. The goal of the interviews was to gather information regarding employment history, work tasks, personal exposure prevention, symptoms commonly associated with lead exposures, and take-home contamination prevention and household composition and to convey health messages.

As of August 31, a total of 233 (65.3%) of 357 workers received at least one BLL test and 185 (51.8%) completed interviews. Among 233 tested workers (median = 16.0 *μ*g/dL; interquartile range = 4.4–30.6 *μ*g/dL), 171 (73.4%) had BLLs ≥5 *μ*g/ dL, 151 (64.8%) had BLLs ≥10 *μ*g/dL, 33 (14.2%) had BLLs≥40 *μ*g/dL, and two (0.9%) had BLLs ≥60 *μ*g/dL. Among 341 household members identified through worker interviews, 46 (13.5%) received a BLL test; none had an elevated BLL. Not all exposed workers and household members were tested for lead, and not every BLL test result might have been reported to WDHS or MDH.

At this time, WDHS and MDH have concluded their joint investigation of the shipyard. The Occupational Safety and Health Administration enforcement investigation began on February 10, 2016 because of lead exposure hazards and revealed that shipyard workers were exposed to lead at ≥20 times the reduced permissible exposure limit of 40 *μ*g/m^3^ (*2*,*3*).

This investigation highlights timely laboratory-based BLL reporting and efficient interstate collaboration. Moreover, it emphasizes the importance of implementing proper engineering controls and periodic BLL monitoring of employees exposed to lead (*4*) and providing correct personal protective equipment for workers in the shipbuilding industry (*3*).
